# Applications of DeepSeek in Medicine: Bibliometric Analysis and Scoping Review

**DOI:** 10.2196/93354

**Published:** 2026-06-15

**Authors:** Haoran Zhang, Dawei Wang, Yanliang Xu, Shuming Han, Guangxin Wang

**Affiliations:** 1 School of Clinical Medicine, Shandong Second Medical University, Weifang, Shandong, China; 2 Shandong Innovation Center of Intelligent Diagnostic Technology, Central Hospital Affiliated to Shandong First Medical University, 105 Jiefang Road, Jinan, Shandong, 250013, China, 86 531 55865152; 3 Key Laboratory of Endocrine Glucose & Lipids Metabolism and Brain Aging, Ministry of Education; Department of Endocrinology, Shandong Provincial Hospital Affiliated to Shandong First Medical University, Jinan, Shandong, China; 4 Library, Shandong Second Medical University, Weifang, Shandong, China

**Keywords:** DeepSeek, large language model, artificial intelligence in medicine, clinical decision support, medical education, scoping review, biomedical ethics, PRISMA

## Abstract

**Background:**

The integration of large language models (LLMs) into medicine has reshaped health care delivery, education, and research. Although proprietary models face challenges such as data privacy, regulation, and adaptability, DeepSeek, an open-source LLM, has emerged as a customizable and cost-effective alternative with significant potential for clinical and operational applications. However, the rapid expansion of research in this area necessitates a systematic mapping of its landscape, applications, and challenges.

**Objective:**

This study combines bibliometric analysis with a scoping review to systematically map and characterize the literature on DeepSeek’s medical applications. The aims were to (1) analyze publication trends, leading contributors, and research themes and (2) identify primary application domains, strengths, limitations, and future directions.

**Methods:**

Following the framework by Arksey and O’Malley and the PRISMA-ScR (Preferred Reporting Items for Systematic Reviews and Meta-Analyses Extension for Scoping Reviews) guidelines, a systematic search was conducted using PubMed, Web of Science, and Scopus from January 20, 2025, to November 30, 2025. Bibliometric analysis was then used to quantify publication trends, productivity, and research themes across 371 papers. The scoping review thematically synthesized the applications, strengths, and limitations of 353 original articles.

**Results:**

The publication output showed a progressive increase, with China (n=163), Turkey (n=52), and the United States (n=48) as leading contributors. Keyword co-occurrence analysis formed 7 clusters; the 3 most frequent keywords were “large language model,” “artificial intelligence,” and “patient education.” DeepSeek has shown promising yet preliminary performance across multiple domains, including patient education, clinical decision support, medical education, workflow optimization, and medical research. The evidence base remains predominantly low in quality, with 66.6% (235/353) of original articles classified as low-quality evidence, consisting largely of unvalidated benchmarking, simulated cases, and single-center retrospective analyses. Only 6.8% (24/353) of studies met the criteria to be considered high quality, and prospective randomized trials assessing patient-relevant outcomes were notably absent.

**Conclusions:**

Publications on DeepSeek’s medical applications increased progressively from January 2025 through November 2025, with China, Turkey, and the United States as the leading contributors. The scoping review found that DeepSeek has been evaluated across 5 domains (patient education, clinical decision support, medical education, workflow optimization, and research), with variable but often competitive performance relative to proprietary models. Strengths included readability, diagnostic accuracy in select specialties, cost-efficiency, and local deployability. Limitations included inconsistent cross-specialty performance, hallucinations, ethical concerns, data privacy issues, and regulatory gaps. The evidence base is predominantly low-quality and simulation-based, with few prospective trials or randomized controlled trials. These findings indicate that DeepSeek’s clinical readiness varies, and future research should address prospective validation, multimodal capabilities, bias mitigation, human oversight, and equitable access.

## Introduction

The integration of artificial intelligence (AI), particularly large language models (LLMs), into medicine has prompted a paradigm shift in health care delivery, education, and research. LLMs, such as OpenAI’s GPT series, have demonstrated considerable capabilities for processing complex medical data, supporting clinical decision-making, and improving patient communication. However, the widespread adoption of proprietary LLMs in clinical settings faces substantial challenges, including data privacy concerns, regulatory constraints, and limited adaptability to institutional requirements [1]. In this context, DeepSeek, an open-source LLM developed by Hangzhou DeepSeek Artificial Intelligence Basic Technology Research Co Ltd, has emerged as a promising alternative, distinguished by its customizability, cost-effectiveness, and alignment with data governance standards [1-3]. This model represents a significant advancement in AI, particularly for its sophisticated reasoning capabilities and its impact on AI research and applications.

DeepSeek’s architecture, especially in reasoning-enhanced iterations such as DeepSeek-R1, incorporates innovative training approaches, including Group Relative Policy Optimization (GRPO). This rule-based reinforcement learning paradigm, which functions without task-specific supervised fine-tuning during the reasoning alignment phase and builds upon a pretrained base model, fosters emergent reasoning behaviors that are particularly valuable for complex medical reasoning tasks [4,5]. This open-weight nature enables local deployment, making it particularly attractive in health care settings where data security and privacy are paramount [1,6]. Since its release, DeepSeek and its associated intelligent agents have been implemented in multiple tertiary hospitals across China, resulting in measurable improvements in clinical and operational workflows, including patient follow-up, imaging analysis, and administrative automation [7-9]. Such real-world implementations underscore the potential for redefining AI-driven health care delivery.

The growing corpus of studies evaluating DeepSeek medical applications has revealed several strengths. In clinical diagnostics, DeepSeek-R1 achieved a diagnostic accuracy comparable to that of GPT-4 in complex clinicopathological cases [10]. In specialized areas, such as ophthalmology, it has exhibited diagnostic and management performance on par with OpenAI o1 while reducing token-related costs by approximately 15-fold [11]. Moreover, DeepSeek excels in Chinese-language medical contexts, outperforming ChatGPT at delivering prostate cancer radiotherapy information in Chinese and demonstrating superior results on Chinese medical licensing examinations [12,13]. Beyond clinical decision support, DeepSeek shows promise in medical education, patient communication, and administrative tasks, with documented deployments across multiple Chinese tertiary hospitals supporting applications ranging from imaging interpretation to automated administrative workflows [9]. However, these promising benchmarking results warrant further examination in real-world clinical settings, which are now emerging primarily in China.

The rapid integration of DeepSeek into clinical practice, particularly within Chinese hospital systems [2,9], underscores the necessity for a thorough evaluation of its applications, limitations, and future directions. The existing literature lacks a comprehensive assessment of publication trends and emerging research fronts in this rapidly evolving domain. Evidence remains fragmented across medical specialties, and the heterogeneous methodologies and outcomes limit a holistic understanding of the model’s clinical utility, safety profile, and readiness for broader implementation. Therefore, a comprehensive synthesis of available evidence is essential to guide health care institutions, policymakers, and developers in evaluating DeepSeek’s realistic capabilities, optimal deployment strategies, and associated risks.

To address this gap and systematically map the research landscape, this study adopted an integrated methodological approach that combined bibliometric analysis with a scoping review. Bibliometric analysis quantitatively characterizes the field at the macro level, examining publication trends over time, core authors and institutions, high-frequency keywords, and journal distributions. This enables the objective identification of research hot spots and evolutionary trajectories [14,15]. Simultaneously, a scoping review is a systematic methodology designed to map key concepts, evidence types, and knowledge gaps within a broad or emerging field. Rather than synthesizing evidence for definitive conclusions, it uses qualitative or descriptive methods to identify existing research themes, methodological characteristics, and underexplored areas, thereby clarifying the overall research landscape [16]. Given that literature on DeepSeek in medicine is growing rapidly and includes highly heterogeneous publications, such as proof-of-concept studies, preclinical research, preliminary clinical trials, and technical descriptions, a scoping review is more suitable than a systematic review for this context, as it focuses on comprehensively mapping the domain without mandating formal quality appraisal. The combination of these two methods leveraged their complementary strengths: Bibliometric analysis provides an objective, structured quantitative overview, while the scoping review delivers a nuanced, contextualized conceptual map. This integrated analysis provided a more powerful and multidimensional understanding of the field’s scope, developmental dynamics, and future directions from both quantitative and qualitative perspectives.

Guided by this integrated approach, the study was structured as follows. First, a bibliometric analysis was conducted to examine relevant original articles and reviews, addressing the following questions: (1) What are the volume, growth trajectory, and geographic distribution of publications? (2) Which countries/regions, institutions, and authors are leading the research? and (3) What are the key research themes and their evolution? Second, a scoping review was performed to critically evaluate the literature content, focusing on the following questions: What are the primary medical application domains of DeepSeek, and how do trends vary across different health care fields? Finally, the discussion synthesizes findings from both methods to highlight implementation challenges, identify major research gaps, and suggest future directions for the effective integration of DeepSeek into global health care systems.

## Methods

### Overview

This study used an integrated approach that combined bibliometric analysis and a scoping review to provide complementary insights. The bibliometric method examined the current application of DeepSeek in medicine from multiple dimensions, analyzed researcher characteristics and journal distributions, and identified research hot spots and trends. The bibliometric analysis was conducted based on the framework proposed by Cobo et al [17], following the guidelines for reporting bibliometric reviews of biomedical literature (BIBLIO) [18]. This scoping review systematically extracted and synthesized the applications, challenges, and future research directions of DeepSeek in medicine. The study was conducted according to the framework by Arksey and O’Malley [19] and reported following the PRISMA-ScR (Preferred Reporting Items for Systematic Reviews and Meta-Analyses Extension for Scoping Reviews) guidelines (Checklist 1) [20].

### Databases, Search Strategy, and Screening Process

To ensure a comprehensive retrieval of the literature on the applications of DeepSeek in medicine, a systematic search was conducted on December 16, 2025, in PubMed, Web of Science Core Collection (WoSCC), and Scopus. The search strategy (Multimedia Appendix 1) used both controlled vocabularies (MeSH, Web of Science Categories, and SUBJAREA) and free-text terms tailored to each database to optimize retrieval.

Given that the public release of DeepSeek’s reasoning model, DeepSeek-R1, on January 20, 2025 [21,22], marked the beginning of subsequent research into its applications, including in medicine, the search encompassed the period from January 20, 2025, to November 30, 2025.

To ensure comprehensive retrieval, the inclusion criteria were as follows: (1) studies investigating the application of DeepSeek in medicine, (2) document types limited to original articles and reviews for bibliometric analysis and original articles only for scoping review, (3) studies published in peer-reviewed academic journals, and (4) no language restrictions.

The exclusion criteria were as follows: (1) duplicate publications; (2) literature that proposed only speculative or hypothetical uses without substantive analysis or findings; (3) non-peer-reviewed journal items, including books, editorials, preprints, commentaries, conference abstracts, case reports, and retracted articles; and (4) studies with insufficient information for bibliometric analysis or whose full text was unavailable for in-depth content extraction during the scoping review.

After receiving professional training, two authors (HZ and DW) independently screened the titles and abstracts and excluded irrelevant studies based on the aforementioned criteria. The interrater agreement was almost perfect (Cohen κ=0.93). Any disagreements during screening were resolved through discussion or, when necessary, arbitration by a third reviewer (GW).

### Bibliometric Analysis

The final bibliometric analysis included 371 papers. Full records of the selected publications were exported and stored in Excel 2021 (Microsoft Corp) and EndNote Desktop (Clarivate). Bibliographic metadata such as authors’ names, affiliations, countries/regions, and keywords were standardized in a uniform format.

Excel 2021 was used to generate tables highlighting the top 10 authors, institutions, and countries/regions based on their publication output, whereas VOSviewer (version 1.6.19) was used for data visualization of bibliometric mapping, including keyword co-occurrence analysis. Keyword co-occurrence analysis examined the fundamental characteristics of keywords, such as their frequency and temporal evolution. This method helped identify research hot spots and track developmental trends within specialized fields. The three common types of visualizations used in the keyword co-occurrence analysis were the network, density, and overlay maps. In the network map, nodes represented keywords, and the connecting lines represented keyword co-occurrence relationships. The size of a node indicates its frequency, the thickness of a line represents the strength of co-occurrence, and the nodes are clustered together by color to reveal distinct research themes or subfields. The overlay map chronologically visualized the keyword trajectories by assigning chromatic codes corresponding to the computationally derived average publication years (APYs). The density map emphasizes the “research density” or concentration of keywords in the knowledge landscape. Areas with numerous closely located keywords appear as warm-colored regions, such as purple, indicating core well-developed research fronts. Cooler-colored areas such as blue or white represent sparser, potentially peripheral, or emerging topics. The centrality of keywords, which reflects their capacity to bridge different parts of the research network, was derived using CiteSpace (version 7.0.0).

### Scoping Review

This scoping review included a total of 353 publications. A data extraction form was created using Excel to extract in-depth content from the papers. This form included items such as paper title, research objectives, key findings, research design types, DeepSeek’s strengths, limitations and challenges, future recommendations, DeepSeek model version, quality tier, and application areas. It should be noted that, although quality assessment is not obligatory for scoping reviews, the methodological quality of all included studies was categorized into 3 tiers (high, moderate, and low) based on the criteria (Multimedia Appendix 2) in order to characterize the strength of the available evidence. Data extraction was conducted independently by 2 authors (HZ and DW). Both authors independently extracted data from all 353 included articles in duplicate using the data extraction form created in Excel. After independent extraction, the 2 authors compared their results. Disagreements were resolved through discussion or by consulting a third author (GW) when consensus could not be reached. The extracted data (Multimedia Appendix 3) were then critically analyzed and organized thematically to address the research question, thereby mapping the key application areas of DeepSeek in medicine. The discussion section elaborates on the challenges, research gaps, and future work for the application of DeepSeek in the medical field.

### Ethical Considerations

Since this study was a bibliometric and scoping review of previously published literature, ethical approval from an ethics committee is not required.

## Results

### Bibliometric Analysis of DeepSeek Applications in Medicine

**Figure 1.**
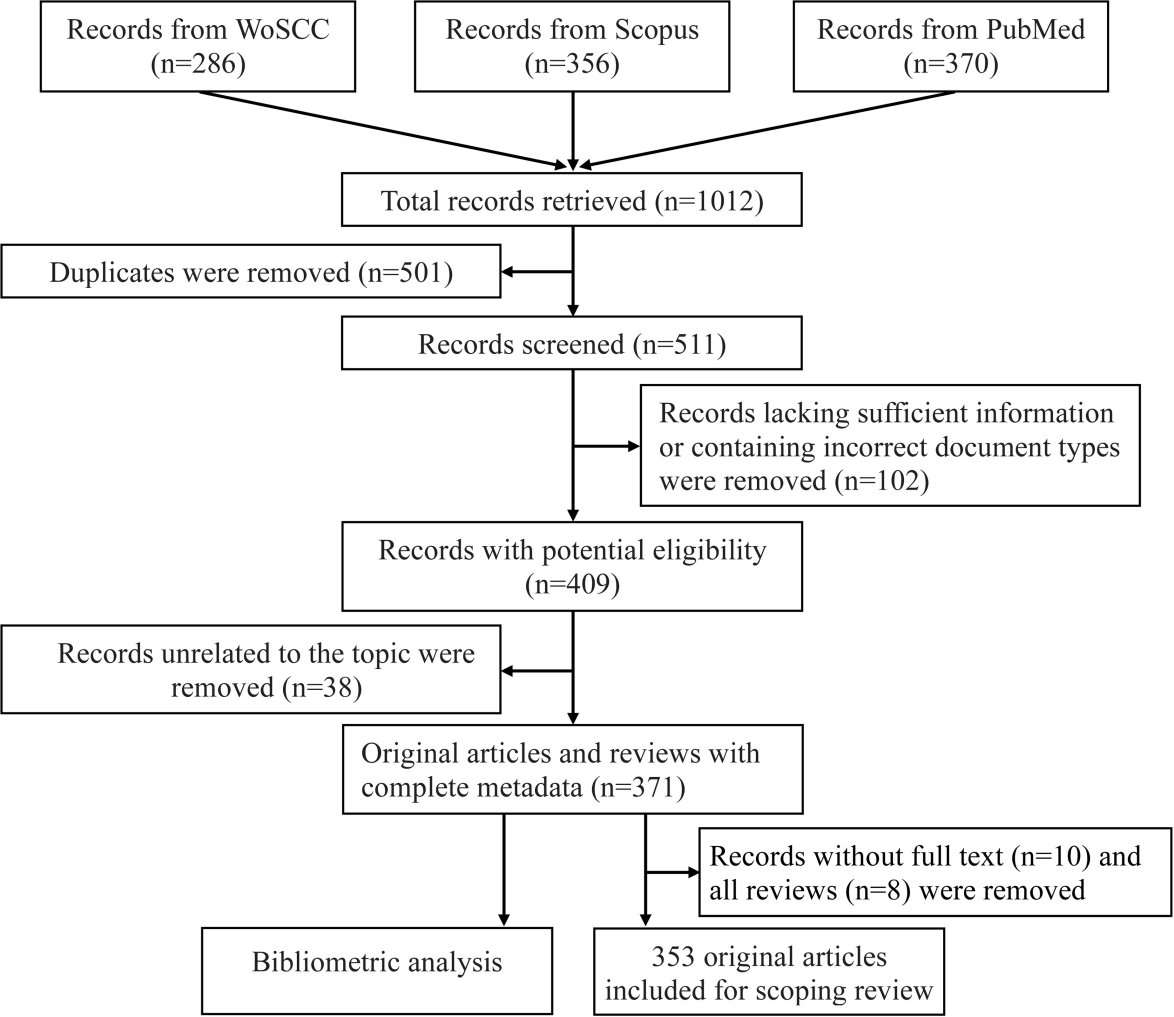
The diagram depicting the paper selection process. WoSCC: Web of Science Core Collection.

A systematic search of PubMed, Scopus, and WoSCC yielded 371 publications on the application of DeepSeek in medicine for bibliometric analysis (Figure 1). Among these, the majority (363/371, 97.8%) were categorized as original articles, while the remaining (8/371, 2.2%) were reviews. In terms of publication languages, 358 papers were written in English, and 13 were written in Chinese.

### Monthly Publication Output

**Figure 2.**
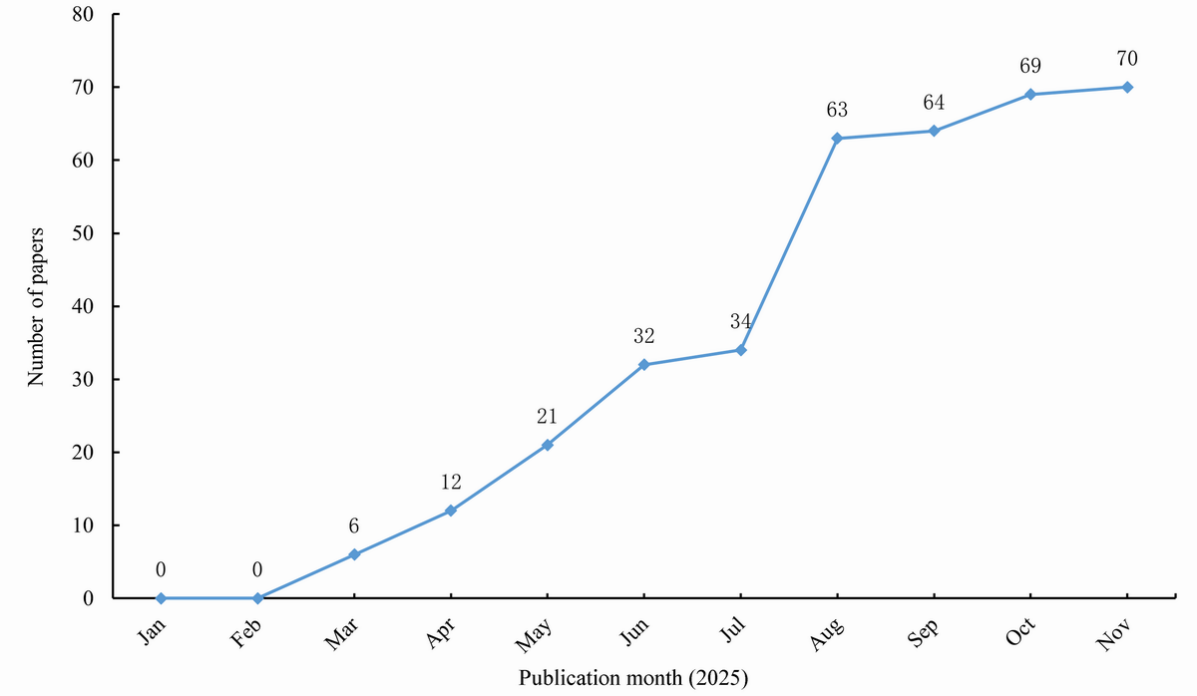
Monthly count of publications on DeepSeek's medical applications identified in this review.

The monthly publication output increased progressively over time. From January to November 2025, the number of papers rose from 0 to 70, with the highest output (70 papers) observed in November (Figure 2).

### Analysis of Source Journals

Of the 216 journals that published papers on the applications of DeepSeek in medicine, 12 published more than 5 papers each. The 10 most active journals collectively contributed 90 publications, accounting for 24.3% (90/371) of the total output. *Cureus* was the most productive journal with 19 publications, followed by *Scientific Reports* (n=10), *BMC Oral Health* (n=9), *International Journal of Medical Informatics* (n=9), *BMC Medical Education* (n=8), *Frontiers in Artificial Intelligence* (n=7), *Frontiers in Public Health* (n=7), *JMIR Medical Informatics* (n=7), *Journal of Medical Internet Research* (n=7), and *Journal of Medical Systems* (n=7).

### The Top 10 Authors, Institutions, and Nations/Regions Ranked by Publication Count

Table 1 presents the top 10 authors, institutions, and countries/regions ranked by their respective number of publications on the applications of DeepSeek in medicine.

**Table 1.** The top 10 authors, organizations, and countries ranked by the number of papers.

Rank	Authors^a^		Organizations^a^		Countries/Regions^a^	
	Name	Papers, n	Name	Papers, n	Name	Papers, n
1	Liu Y	6	Shanghai Jiao Tong University	16	China	163
2	Zhang J	5	Chinese Academy of Medical Sciences	10	Turkey	52
3	Li J	5	Sichuan University	10	United States	48
4	Wang J	5	Zhejiang University	9	Germany	24
5	Wang Y	5	Capital Medical University	9	India	23
6	Xu L	4	University of Health Sciences, Turkey	9	United Kingdom	20
7	Rozen WM	3	Southern Medical University	8	Italy	14
8	Cuomo R	3	Soochow University	7	Saudi Arabia	14
9	Marcaccini G	3	Sun Yat-sen University	7	Australia	9
10	Chen S	3	Tsinghua University	6	Canada	8

a These 3 categories are independent of each other.

### Most Cited Papers on the Medical Applications of DeepSeek

Table 2 lists the 10 most-cited publications on the medical applications of DeepSeek: 9 were original articles, while 1 was a review [12,23-31].

**Table 2.** Top 10 most-cited publications on the medical applications of DeepSeek.

Rank	Authors	Publication date	Total citations, n	Research focus
1	Zhou et al [23]	June 2025	50	Comparative evaluation of DeepSeek and ChatGPT models
2	Deng et al [24]	May 2025	38	DeepSeek’s advances, applications, and challenges across various domains, including health care
3	Kaygisiz and Teke [25]	April 2025	29	DeepSeek’s diagnostic performance in oral pathologies
4	Rasool et al [26]	March 2025	28	DeepSeek’s emotion-aware embedding fusion for responses
5	Yilmaz et al [27]	April 2025	16	Comparative performance of LLMs^a^ on oral pathology multiple-choice questions
6	Marcaccini et al [28]	March 2025	16	DeepSeek and AI^b^ in hand fracture management
7	Luo et al [12]	April 2025	16	DeepSeek versus ChatGPT in multilingual prostate cancer radiotherapy
8	Özcivelek and Özcan [29]	May 2025	15	Comparative evaluation of AI chatbots on dental and maxillofacial prostheses
9	Gültekin et al [30]	August 2025	14	Comparative evaluation of AI models for patient education
10	Seth et al [31]	March 2025	12	Evaluating DeepSeek and AI in hand surgery decisions

a LLMs: large language models.

b AI: artificial intelligence.

### Keyword Co-Occurrence Analysis

**Figure 3.**
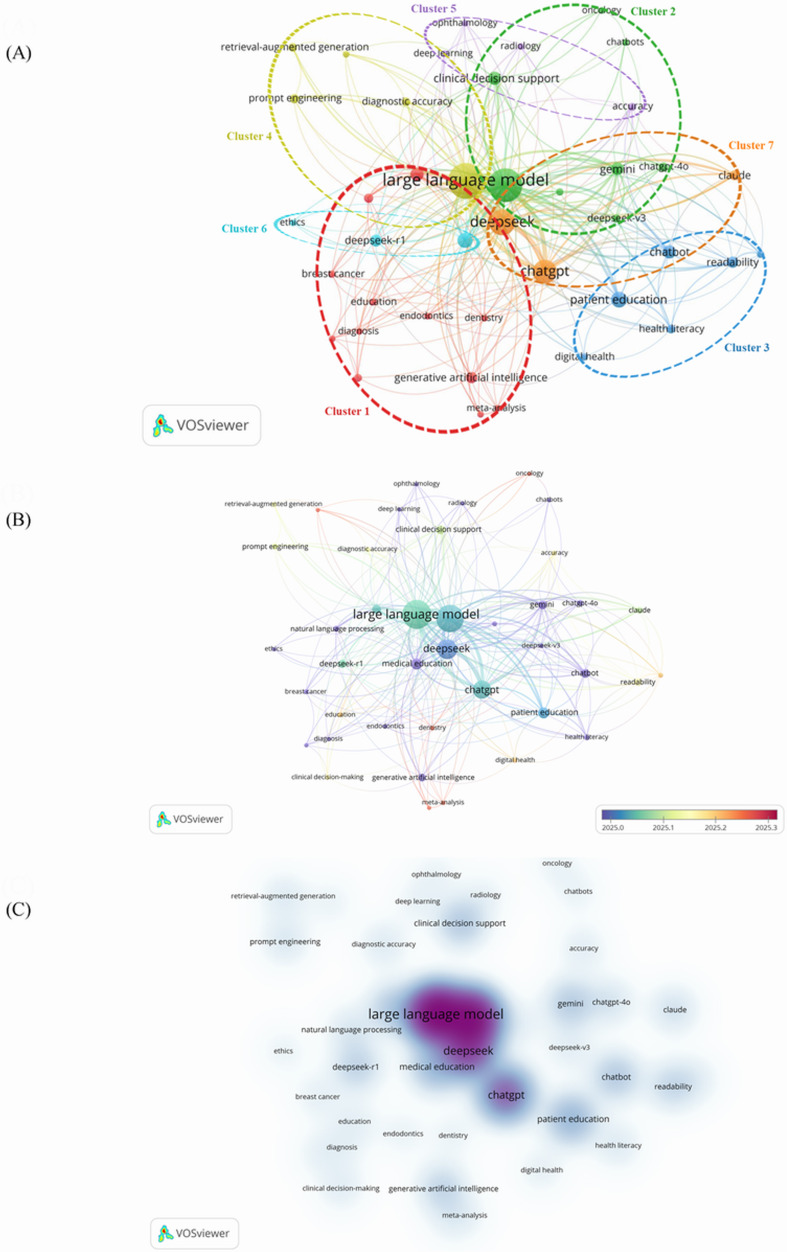
Keyword co-occurrence analysis visualization using VOSviewer [32,33]: (A) network visualization, with keywords grouped into 6 distinct thematic clusters; (B) overlay map colored by the average publication year of each keyword, ranging from purple (earlier) to crimson (recent); and (C) density map based on keyword co occurrence frequency, where color intensity reflects occurrence rate: crimson (highest), blue (moderate), and white (lowest).

A keyword co-occurrence analysis was performed to map predominant research hot spots. Synonyms were consolidated prior to analysis; specifically, “large language model(s)” was standardized as “large language model,” and “generative artificial intelligence/AI” was standardized as “generative artificial intelligence.” The top 10 keywords by frequency are listed in Table 3. Notably, “generative artificial intelligence” ranked seventh in frequency but third in centrality. From an initial set of 968 keywords, 41 occurring more than 4 times were included in the keyword co-occurrence analysis. These formed 7 well-defined clusters, visualized in the network map (Figure 3A).

The temporal overlay map (Figure 3B) illustrates the evolution of research focus, with keywords colored by their APYs. Purple nodes represent earlier themes, while crimson indicates more recent activity. Early research concentrated primarily on medical education. The keywords “retrieval-augmented generation” and “oncology” showed the highest APY, reflecting a rising interest in these areas.

The density map (Figure 3C) displays keywords according to their average frequency of occurrence. Crimson regions correspond to the most frequently occurring keywords, followed by blue and then white areas, in descending order.

**Table 3.** The top 10 keywords regarding DeepSeek’s applications in medicine.

Rank	Keywords	Frequency of occurrence, n	Centrality
1	Large language model	227	1.00
2	Artificial intelligence	197	0.55
3	Patient education	30	0.02
4	Medical education	28	0.01
5	Clinical decision support	19	0.01
6	Machine learning	19	0.05
7	Generative artificial intelligence	19	0.07
8	Natural language processing	9	0.01
9	Prompt engineering	8	0.00
10	Diagnostic accuracy	8	0.03

#### Summary of Extracted Data in the Scoping Review: Study Quality, Model Versions, Comparative Performance, and Documented Limitations

Of the 353 original articles, 24 (6.8%) met the criteria for high quality. These were primarily prospective evaluations and studies with external validation. A further 94 studies (94/353, 26.6%) were classified as moderate quality. The majority (235/353, 66.6%) were classified as low quality, reflecting the exploratory nature of the current evidence base, which is dominated by invalidated benchmarking using examination questions and single-center retrospective analyses.

Analysis of DeepSeek-specific versions revealed that DeepSeek-R1 was the most frequently studied (mentioned in 197 papers, 55.8% of the 353 articles), followed by DeepSeek-V3 (114/353, 32.3%) and unspecified versions of DeepSeek (61/353, 17.3%).

A total of 283 studies compared DeepSeek with other LLMs, primarily ChatGPT, in medical applications. Among these, 126 studies (126/283, 44.5%) reported positive results in which DeepSeek outperformed or showed significant advantages; 84 studies (84/283, 29.7%) reported neutral results with comparable performance, no statistically significant difference, or mixed strengths and limitations; and 73 studies (73/283, 25.8%) reported negative results in which DeepSeek underperformed relative to other models.

DeepSeek’s primary weaknesses included inconsistent domain performance in 61 papers, incomplete answers in 47 papers, poor readability in 42 papers, and hallucinations in 38 papers. Ethical risks, though fewer in absolute count at 57 papers, were severe; specifically, non-maleficence was documented in 22 papers with potential patient harm, autonomy was documented in 15 papers with privacy and informed consent concerns, beneficence was documented in 8 papers with lack of empathy and impaired therapeutic relationship, and justice was documented in 12 papers highlighting bias and inequity. Other barriers reported in 55 papers further hindered clinical adoption.

#### Application Domains of DeepSeek in Medicine

Based on the scoping review of 353 full-text papers, the medical applications of DeepSeek can be summarized into the primary domains discussed in the following sections. Because a single study often evaluated DeepSeek in multiple domains, the sum of article counts across these domains exceeds 353.

##### DeepSeek in Patient Education and Communication

The applications of DeepSeek in patient education and communication were addressed in 105 articles. Among these, 91 were cross-sectional studies, 5 were descriptive studies, 4 were prospective studies including 1 randomized controlled trial (RCT), and the remaining 5 used other design types.

DeepSeek can generate patient-facing materials that are both readily comprehensible and clinically accurate. This capability has been empirically validated; for example, in generating patient education materials for spinal surgeries, DeepSeek-R1 achieved the lowest Flesch-Kincaid Grade Level scores, indicating content accessible to a broader audience including those with limited health literacy [23]. Similarly, in orthopedics, DeepSeek-R1 provided clearer and more easily understandable explanations of anterior cruciate ligament surgery than ChatGPT, which offered greater comprehensiveness but at a higher reading level [30]. This emphasis on linguistic accessibility is critical in patient-facing materials because improved readability enhances patient engagement, reduces anxiety, and supports informed decision-making [23,34]. Furthermore, DeepSeek has performed strongly in multilingual contexts, effectively generating patient education content in both Chinese and English, which is vital for serving diverse linguistic populations [12,35].

Although DeepSeek excels in readability, its responses sometimes lack comprehensive detail or sufficient citations of sources, and occasional inaccuracies or AI hallucinations have been noted [29,36,37]. Furthermore, some studies found that DeepSeek performed similarly to, or even less accurately than, ChatGPT when generating patient education materials [38,39].

##### DeepSeek in Clinical Decision Support and Treatment Planning

Of the 176 articles addressing DeepSeek in clinical decision support and treatment planning, 120 were cross-sectional studies, 22 were retrospective studies, 9 were prospective studies (including 2 RCTs), 2 were mixed-design studies, 14 were proof-of-concept studies, and the remaining 9 articles comprised expert consensus and other designs.

Regarding diagnostic accuracy, DeepSeek models have achieved notable results. In a dual-phase retrospective-prospective study classified as high methodological quality (n=300 liver lesions in the retrospective cohort and 126 liver lesions in the prospective cohort), DeepSeek-V3 demonstrated higher Liver Imaging Reporting and Data System (LI-RADS) classification accuracy than junior radiologists and achieved performance comparable with that of senior radiologists for hepatocellular carcinoma diagnosis [40]; however, this finding awaits replication in larger, multicenter settings. In a moderate-quality historical control study, DeepSeek-R1 demonstrated diagnostic accuracy comparable to that of GPT-4 in complex clinicopathologic cases [10]. In a low-quality cross-sectional study, Jiao et al [11] found that diagnostic accuracy in diagnosing corneal diseases varied significantly among LLMs (*P*=.001). GPT-4o achieved the highest accuracy (80%), while DeepSeek R1 achieved only 65%; both had accuracies that were significantly lower than that of human experts (92.5%; (*P*<.001).

For treatment planning, DeepSeek-V3 demonstrated statistically superior accuracy compared with ChatGPT-o1 in head and neck cancer management [41], and DeepSeek-R1 outperformed OpenAI o1 in diagnostic accuracy and next-step decision-making in ophthalmology [42]. These models have demonstrated strengths in specialized domains, including hand fracture management [28], urinary incontinence management [43], and postprostatectomy urinary incontinence guidelines [44], although they have limitations in complex scenarios. Notably, DeepSeek’s clinical reasoning capabilities are enhanced through its reinforcement learning framework, which enables emergent reasoning patterns, such as self-reflection and verification [5], contributing to its strong performance in clinical decision support tasks. However, although DeepSeek shows promising capabilities for clinical decision support, it cannot replace multidisciplinary tumor boards or human expertise, as it lacks contextual clinical judgment, physical examination capabilities, and the ability to negotiate complex trade-offs among specialists; instead, it streamlines clinical workflows by rapidly organizing patient data [41]. The integration of few-shot prompting has been shown to substantially enhance DeepSeek’s accuracy in specialized tasks, such as Coronary Artery Disease Reporting and Data System (CAD-RADS) category assignment [42], suggesting that optimal prompt engineering is crucial for clinical implementation.

Overall, DeepSeek has emerged as a scalable tool to support treatment decisions, streamline workflows, and reduce diagnostic errors; however, integration requires careful validation and human oversight to mitigate risks.

##### DeepSeek in Medical Education and Benchmarking

Of 109 articles addressing the applications of DeepSeek in medical education and benchmarking, 93 were cross-sectional studies, 6 were retrospective studies, 5 were perspective studies, and 5 were descriptive studies.

On the Chinese National Medical Licensing Examination, DeepSeek-R1 achieved 92% accuracy, significantly outperforming ChatGPT-4o (87.2%) and demonstrating strength on low-difficulty questions [13]. Similarly, in the gastroenterology board examinations, both the base R1 model (77.1%) and search-augmented version (81.5%) surpassed the passing threshold and significantly outperformed the offline ChatGPT-3 (65.1%) and ChatGPT-4 (62.4%) models [45]. Cross-specialty comparisons revealed consistent patterns: In basic medical sciences, DeepSeek-R1 scored 78.33% alongside ChatGPT-4, whereas in clinical sciences, it scored 87.5%, demonstrating robust knowledge integration [46]. When evaluated against other reasoning-enhanced models on ophthalmology board-style questions, DeepSeek-R1 (72.5%) and its lighter variant R1-Lite (76.5%) performed competitively with OpenAI o1 Pro (83.4%), suggesting a balanced trade-off between performance and computational efficiency [47]. The model also demonstrated strong anatomical knowledge, achieving 89.2% accuracy on Turkish Dental Specialty Admission Exam anatomy questions, comparable with other major models, though below ChatGPT-4o’s 98.6% [48]. These benchmark studies collectively indicate that DeepSeek provides a cost-effective, open-weight alternative for medical education, with utility in knowledge assessment and examination preparation. However, performance gaps persist in specialized domains and image-based questions, highlighting areas for future development and the continued need for human oversight in comprehensive medical education frameworks.

##### DeepSeek for Clinical Workflow Optimization

A total of 63 articles described DeepSeek for clinical workflow optimization, including 26 cross-sectional studies, 2 descriptive studies, 17 retrospective studies, 4 prospective studies, 10 proof-of-concept studies, and 4 articles with other study designs.

The integration of DeepSeek models into health care systems offers significant potential to enhance operational efficiency and streamline clinical workflows, primarily by automating routine and time-consuming tasks. A prominent example is the locally deployed closed-loop system powered by DeepSeek for quality control of electronic nursing documentation. This system implements a comprehensive framework spanning the real-time, final, and vertical dimensions of quality assurance. The results include a dramatic reduction in documentation omission rates from 7.19% to just 1.79%; a decline in logical inconsistencies from 9.35% to 0.72%; and the complete elimination of timeliness errors, which previously stood at 8.63%. Concurrently, the quality control time per record decreased by 3.2-fold, reallocating nursing efforts toward direct patient care [6].

In dyslipidemia management, DeepSeek, alongside Claude-3 and GPT-4, optimized guideline-based workflows across 30 standardized cases, boosting accuracy from 72% for physicians to 91% with AI. Integration with human experts further raised simulated low-density lipoprotein cholesterol target attainment to 92%, demonstrating its utility in minimizing guideline deviations while enhancing workflow efficiency [49]. However, one moderate-quality study found that DeepSeek R1 achieved an accuracy of only 48.4% in a noncritical emergency department triage task, which is significantly lower than that of another LLM, Gemini 2.0 flash (73.8%) [50].

The large-scale deployment of DeepSeek across nearly 90 Chinese tertiary hospitals has reportedly increased patient follow-up efficiency 40-fold, marking a transformative impact on hospital administration and clinical workflow automation [9]. By managing labor-intensive tasks with high consistency and speed, DeepSeek enables a paradigm shift from reactive to proactive operational governance. This transition enabled health care professionals to focus their expertise on more complex clinical decision-making responsibilities.

### Medical Research and Data Analysis

Medical research and data analysis were mentioned in 73 articles. Among these, 41 had a cross-sectional design, 6 were descriptive studies, 2 were perspective studies, 9 were proof-of-concept studies, 9 were retrospective studies, 1 had a mixed design, and the remaining 5 used other design types.

DeepSeek models have demonstrated significant utility in accelerating and refining medical research and data analysis workflows. DeepSeek facilitates the reading of medical literature, information extraction, and screening. Several studies have developed AI-powered screening tools using DeepSeek to identify relevant studies for systematic reviews, reporting high accuracy and a significant reduction in manual workload [51-53]. For example, the LitAutoScreener tool, which integrates DeepSeek, achieved high accuracy and significantly improved screening efficiency, reducing the processing time to seconds per article [51]. Similarly, other evaluations have confirmed that DeepSeek-based tools can reduce manual workload while maintaining high recall rates in literature screening for meta-analyses [53]. In fields such as aging research, DeepSeek-R1 is part of a multi-LLM ensemble that successfully extracts protocol details from clinical trial records, doubling the yield of conventional search methods and achieving expert-level accuracy for core data points [54]. Second, DeepSeek assists with generating and refining research topics and study designs. It helps researchers analyze cutting-edge trends, funding guidelines, and successful grant applications, thereby validating the novelty of the proposed research questions [3]. For instance, DeepSeek-R1 has been used to explore novel research ideas and generate systematic review topics in fields such as oral and maxillofacial surgery [55]. Similarly, in biomedical research, DeepSeek models show promise in extracting structured pre-analytical variability data from the scientific literature, facilitating standardized reporting and systematic evaluation [56]. Furthermore, DeepSeek serves as a valuable tool for peer review and for critiquing research proposals. Its capacity to generate high-quality evidence-based responses enables a preliminary assessment of a proposal’s feasibility and soundness. This function is particularly beneficial in multidisciplinary contexts where the model’s ability to synthesize information from diverse sources significantly enhances the evaluation process [57,58]. Third, DeepSeek demonstrated substantial potential as an assistant for drafting, editing, and refining the content of medical research papers. Its capabilities span various domains of medical research and practice, making it a versatile tool for enhancing the quality and efficiency of academic writing. The model’s proficiency at generating structured, clear, and comprehensible content is particularly valuable in medical research, where precision and clarity are paramount [59].

### Other Application Domains

In other application domains, 25 articles were identified, comprising 18 cross-sectional studies, 2 perspective articles, 2 descriptive studies, and 3 proof-of-concept studies.

Beyond the primary domains discussed, DeepSeek has been explored in several niche but critical areas, including treatment outcome prediction, drug development assistance, and suicide risk prediction. Instead of reactive question-answering, DeepSeek is integrated into predictive analytics platforms. It can proactively flag at-risk patients, suggest personalized screening intervals, and predict individual responses to therapies based on electronic health records and real-time data [60,61]. In nasopharyngeal carcinoma, DeepSeek-V3-0324 demonstrated superior performance in treatment response evaluation compared with ChatGPT-4o-latest (96.5% vs 82.9%) and showed stronger agreement with expert annotations [62].

In drug discovery, DeepSeek aids with predicting drug-drug interactions and molecular property modeling, achieving superior performance in regression and classification tasks critical to drug discovery [63,64].

The model’s chain-of-thought enabled analysis of factors associated with correct predictions, such as substance abuse and age-related comorbidities. This application underscores DeepSeek’s potential for mental health risk assessment, though further validation is needed [65].

## Discussion

### Main Findings

This integrated bibliometric and scoping review provided a comprehensive early-stage mapping of the rapidly evolving research landscape concerning DeepSeek’s applications in medicine. This field is characterized by explosive growth, global engagement, and exploration across a remarkably diverse spectrum of clinical and operational domains. The findings collectively underscore DeepSeek’s emergence not merely as another LLM but as a potent, open-weight contender with specific capabilities that address critical needs in modern health care, including cost-effectiveness, linguistic accessibility, and scalability.

Bibliometric data showed a research frontier that has been intensively explored. The increased publication output regarding applications of DeepSeek in medicine is clear. Our results align with those of an analysis of the global research profile of another LLM, ChatGPT, conducted by Alessandri-Bonetti et al [66], who revealed explosive growth in publications during the first 7 months after its release. This pattern is also consistent with a broader LLM systematic review by Chen et al [67], which reported that, between January 2022 and September 2025, approximately 3.2 clinical LLM studies were published per day, with a linear increase of 7.04 studies per month following the release of ChatGPT. Notably, DeepSeek was not included in the analysis by Chen et al [67], underscoring the gap and the need for our focused review.

The geographical and institutional productivity led by China, followed by Turkey and the United States, reflects widespread international interest of DeepSeek’s potential, with major academic medical centers driving early investigations. Papers on DeepSeek’s applications in medicine have been published in various journals, ranging from well-known open-access journals such as *Cureus* and *Scientific Reports* to professional medical informatics and medical education journals such as the *Journal of Medical Internet Research*. This publication pattern indicates that the research reaches both broad scientific and specialized clinical audiences. Keyword co-occurrence analysis effectively identified the core themes of this research trend. The temporal overlay, which revealed a shift from foundational medical education topics toward more specialized areas such as “retrieval-augmented generation” and “oncology,” illustrates the field’s rapid maturation and deepening focus. Synthesizing the scoping review findings, DeepSeek as a medical tool initially gained attention for its strength in democratizing medical information. For instance, in patient education, it can generate outputs with higher readability than its counterparts, such as ChatGPT.

Perhaps the most striking finding is that DeepSeek has demonstrated competitive and sometimes superior performance compared with existing proprietary models in clinical decision support tasks. The bibliometric analysis revealed that “clinical decision support” formed the largest cluster, while the scoping review further indicated that these studies primarily focused on three specific tasks: “aiding diagnosis,” “differential diagnosis,” and “treatment plan formulation.” The evidence that DeepSeek-V3 can match senior radiologists at specialized diagnostic classifications or that DeepSeek-R1 rivals GPT-4 and OpenAI o1 in diagnostic accuracy across ophthalmology and complex clinicopathological cases challenges the assumption that superior capability is the exclusive domain of closed, commercial models. This “performance parity” achieved through an open-weight architecture has profound implications. Specifically, it suggests a pathway toward breaking the monopoly of advanced AI in clinical support, potentially fostering innovation, reducing costs, and allowing for better adaptation to local health care contexts and linguistic needs.

The utility of this model in medical education and benchmarking further supports its position as a disruptive and cost-effective tool [68]. For institutions and learners worldwide, particularly in resource-constrained settings, DeepSeek offers a viable, high-quality alternative for exam preparation, simulation, and curriculum development, potentially lowering the barriers to accessing advanced medical training aids.

Beyond its direct clinical and educational applications, this review highlighted DeepSeek’s transformative potential across broader health care operations. Documented case studies have demonstrated reductions in documentation error rates in nursing and lower specimen return rates in gynecological examinations and enabled large-scale patient follow-up. By automating a vast array of low-complexity tasks, DeepSeek can free human resources to provide higher quality care and reduce systemic inefficiencies across the health care continuum.

Of the 353 papers included in this scoping review, only 6.8% (24/353) met the criteria for high quality, whereas the majority (235/353, 66.6%) were classified as low quality, consisting predominantly of invalidated benchmarking using examination questions, single-center convenience samples, and proof-of-concept studies. This distribution reflects a critical gap in the current literature: The rapid proliferation of DeepSeek in medicine has been accompanied by an abundance of exploratory studies with limited external validity. Although such benchmarking studies offer valuable insights into the model’s technical capabilities and serve as initial performance indicators, they do not directly inform real-world diagnostic accuracy, patient safety, or clinical utility [69].

In head-to-head comparisons with other LLMs, DeepSeek demonstrated predominantly favorable or comparable performance: Positive outcomes (126/283, 44.5%) were more frequent than negative ones (73/283, 25.8%), and a substantial proportion of studies (84/283, 29.7%) showed no clear superiority of either model. However, because these results derived predominantly from low-quality (235/353, 66.6%) or moderate-quality evidence, with only 6.8% (24/353) meeting high methodological standards, performance claims should be considered preliminary and hypothesis-generating rather than definitive. Clinically, it excels in open-source accessibility, low cost, readability, Chinese language proficiency, and structured reasoning; nonetheless, limitations, including occasional inaccuracies, lower reliability in certain tasks, and the absence of prospective clinical trials, necessitate continued validation and human oversight.

### Comparison With Prior Reviews on Other LLMs in Medicine

To contextualize the novel and distinct contributions of our work, we compared this review with existing reviews of other LLMs in medicine, such as ChatGPT, GPT-4, LLaMA, and Gemini. Several prior reviews have documented the rapid adoption of proprietary LLMs in health care, highlighting their utility in clinical reasoning, medical education, and patient communication [66,70,71]. However, most existing reviews have primarily focused on closed-source models, which are characterized by limited transparency, restricted capacity for local deployment, and substantial cost barriers. These limitations hinder their scalability and reduce their adaptability across diverse institutional settings. In contrast, this review specifically focused on DeepSeek, an open-weight LLM, and identified several distinctive features that differentiate it from the patterns reported in previous LLM reviews.

First, methodologically, we combined bibliometric analysis with a scoping review to provide both quantitative mapping of research trends and qualitative synthesis of applications and challenges of DeepSeek in medicine, a dual approach rarely applied in prior LLM reviews, which have tended to rely on either bibliometric or narrative synthesis alone [66,71].

Second, geographically, the research landscapes differ substantially. For ChatGPT, early publications were predominantly led by institutions in the United States and Europe, with a wide distribution across high-income countries [66,70]. In contrast, our analysis identified China as the dominant contributor to DeepSeek medical research (163 papers), followed by Turkey and the United States. This pattern aligns with DeepSeek’s country of origin and its rapid deployment across Chinese tertiary hospitals [2,9]. Notably, the early and substantial involvement of Turkish researchers (52 papers) in DeepSeek research is a distinctive feature not observed in early ChatGPT literature.

Third, previous reviews focused predominantly on proprietary models such as ChatGPT, GPT-4, LLaMA, and Gemini. In contrast, our study addressed a significant gap by examining an open-source alternative with distinct architectural advantages and greater deployment flexibility. In terms of real-world deployment, the deployment of DeepSeek across nearly 90 tertiary hospitals in China has resulted in measurable improvements in workflow efficiency and documentation quality. This scale of implementation has not been reported in similar reviews of other LLMs, which have largely focused on simulated or benchmarking studies [6,9]. In terms of application areas, prior work on ChatGPT and other proprietary LLMs identified medical education, clinical decision support, and patient communication as core areas [70,71]. Our keyword co-occurrence analysis confirmed that these are also central themes for DeepSeek. However, DeepSeek’s open-weight architecture introduces distinctive features not emphasized in proprietary LLM reviews: on-premises deployability, data privacy, cost-effectiveness, and superior performance in Chinese-language medical tasks. These features represent unique contributions of DeepSeek to the medical LLM landscape and are not simply typical of any newly introduced LLM. Regarding performance and utility, our findings demonstrated that DeepSeek achieved competitive or superior performance compared with proprietary models in clinical diagnostics, medical licensing examinations, and patient education while substantially reducing costs, advantages that prior reviews have identified as critical unmet needs in AI integration [71,72].

### Challenges in the Applications of DeepSeek in Medicine

Guided by an ethical framework, the efficacy and safety of any medical intervention must be carefully calibrated in modern medical practice [73]. As aforementioned, DeepSeek demonstrates significant potential for enhancing medical workflows, medical education, and research. However, its application faces numerous challenges in terms of effectiveness and safety, including accuracy issues, data privacy concerns, ethical uncertainties, and diverse global regulations governing AI.

### Accuracy and Variable Performance Across Medical Domains and Specialties

Although DeepSeek has demonstrated diagnostic accuracy comparable to that of specialist clinicians and proprietary models in certain areas [40,74-76], its overall efficacy remains inconsistent [42,77,78]. The model exhibits strong zero-shot and few-shot learning capabilities in general tasks; however, the rapid evolution of medical knowledge necessitates continuous pretraining on extensive volumes of high-quality, domain-specific data. In data-scarce specialties, particularly those lacking sufficient fine-tuning datasets, DeepSeek often fails to effectively acquire new features and patterns, leading to model hallucinations, defined as the generation of seemingly plausible but factually incorrect or unsupported information [36,79]. Such limitations are particularly severe in domains involving rare diseases and complex, nonclassical clinical scenarios, where available pretraining data are often insufficient and clinically unvalidated [36,37,80,81]. Furthermore, as a fundamentally text-based model, DeepSeek exhibits inherent limitations in processing specialized nontextual medical data, such as medical images, complex laboratory metrics, and genomic data [74,82-84]. These constraints collectively contribute to inconsistent model performance across specific medical domains and hinder its generalization.

### Ethical and Safety Risks

The integration of DeepSeek into medical practice raises ethical challenges that implicate all 4 foundational principles of biomedical ethics, namely autonomy, nonmaleficence, beneficence, and justice, which were originally proposed by Beauchamp and Childress in 1979 [85].

#### Autonomy: Challenges to Patient Self-Determination and Informed Consent

The application of DeepSeek in medicine may undermine the principle of autonomy in medical ethics. As an open-source model, DeepSeek can be deployed on-premises in a hospital environment, which facilitates compliance with data privacy requirements [1,9,81]. However, its broader adoption is complicated by varying regulatory frameworks across regions, such as the General Data Protection Regulation (GDPR) and the Health Insurance Portability and Accountability Act (HIPAA) [3]. The Italian data protection authority, for instance, has restricted DeepSeek over concerns that its data handling methods fail to meet the strict privacy rules of the European Union [81]. Although techniques such as chain-of-thought have enhanced the interpretability of decision-making, the model’s fundamental “black-box” nature persists, posing practical challenges to informed consent in clinical applications [60,86-90].

#### Nonmaleficence: Risks of Novel and Amplified Harms

The rapid, cost-effective integration of DeepSeek in Chinese hospitals underscores a central paradox in medicine: how to seize the opportunity for transformative innovation while mitigating the risks of undue haste and still upholding the principle of “first, do no harm” [2]. However, this model may provide overly definitive recommendations, potentially suggesting unnecessary tests or harmful treatments without adequate contextual warnings [91,92]. If clinicians over-rely on AI outputs, effectively delegating core cognitive tasks such as comprehensive analysis, differential diagnosis, and clinical judgment to the machine, it may lead to the erosion of clinical skills and their independent clinical reasoning. Furthermore, however data-driven its suggestions may be, DeepSeek may lack the nuanced and holistic understanding of a patient’s psychosocial context that an experienced physician integrates. Collectively, these issues challenge the ethical principle of nonmaleficence.

#### Beneficence: The Challenge of Defining and Delivering “Good”

The principle of beneficence obligates health care providers to act in ways that promote patients’ well-being and enhance clinical outcomes [93]. However, an emphasis on AI-driven efficiency may unintentionally marginalize the irreplaceable human dimensions of medicine, such as empathy, compassion, and the therapeutic physician-patient relationship. Although systems like DeepSeek are adept at optimizing measurable, data-informed endpoints, the concept of “good” in medical practice encompasses psychosocial, spiritual, and qualitative aspects of care that resist easy quantification [89,94]. Overreliance on algorithmic pathways designed to maximize metrics neglects the holistic components of beneficence [95]. Consequently, the physician’s role as a compassionate interpreter of illness, which lies at the heart of medical beneficence, may be subordinate to the pursuit of algorithmic efficiency.

#### Justice: Amplifying Inequities in Algorithmic Health Care

The principle of justice concerns fair and equitable distribution of health care benefits and burdens. Despite the use of data preprocessing techniques and fairness-aware algorithms, DeepSeek can still perpetuate and potentially amplify societal or health care biases present in its historical medical training data, including the underdiagnosis of certain conditions within specific demographic groups, thereby harming marginalized populations [80,88,96]. Furthermore, because DeepSeek’s training framework is primarily optimized for English and Chinese, it carries inherent lexical and cultural biases that may limit its applicability to global health care contexts [12,35,97]. Additionally, the benefits of advanced AI, such as DeepSeek, are likely to accrue disproportionately to well-resourced tertiary-care urban hospitals equipped with the necessary infrastructure and specialized personnel for local deployment. Such unequal access exacerbates existing health disparities across regions and socioeconomic groups.

### Other Challenges

In addition to challenges such as accuracy, variable performance across medical domains and specialties, and medical ethics and safety issues, the application of DeepSeek in medicine faces other obstacles, including the redesign of clinical workflows, delineation of liability, regulatory lag, and trust and adoption. The deployment of DeepSeek challenges some clinicians’ work habits and creates a demand for professionals who understand both clinical practice and AI. A shortage of talent limits its wider adoption. When errors in DeepSeek-assisted decision-making lead to medical incidents, how should legal responsibility be defined? Should it fall on the operating physician, the hospital that adopted the AI, or the model developers? Currently, global regulations in this field generally lag, and this uncertainty greatly dampens hospitals’ willingness to implement such technologies. Trust remains another challenge; although DeepSeek is easy to use, concerns about risks affect its acceptance [87].

### Future Work in the Applications of DeepSeek in Medicine

Based on the aforementioned challenges, future research and development should prioritize the directions highlighted in the following sections to advance the reliable, ethical, and equitable integration of DeepSeek into medical practice.

#### From Benchmarking to Clinical Validation: Prospective and Pragmatic Studies

The current evidence base is dominated by low-quality, simulation-based studies. Future work should move beyond examination-style benchmarks and retrospective analyses toward prospective, multicenter, and pragmatic clinical trials. Specifically, RCTs are urgently needed to compare DeepSeek-assisted care against standard practice using both proximal performance metrics, such as diagnostic accuracy, and patient-relevant outcomes, including treatment adherence, adverse events, and quality of life [98,99]. Such trials should also evaluate human-AI interaction models, for example, human-in-the-loop versus fully automated approaches, to determine the optimal balance between efficiency and safety [100,101]. Furthermore, real-world implementation science frameworks should be applied to assess scalability, usability, and unintended consequences across diverse health care settings.

#### Strengthening Governance, Explainability, and Safety

To address ethical and regulatory gaps, future work should co-develop clinically interpretable explainability methods tailored to DeepSeek’s reasoning architecture. Techniques such as structured audit trails, uncertainty quantification, and natural language rationales can support informed consent and clinician oversight [89,102]. On the governance front, clear liability and accountability frameworks are required to delineate responsibilities among developers, health care institutions, and clinicians when AI-assisted errors occur [88,96]. Additionally, the “human-in-command” principle, which mandates that DeepSeek’s recommendations serve as decision support rather than replacement for clinician judgment, should be embedded into clinical workflows and professional guidelines [98,103]. As articulated in the concept of AI-assisted medicine introduced by Wang et al [104], a discipline that uses AI technologies to assist with disease research, prevention, diagnosis, and treatment as well as to promote health maintenance, clinicians must retain ultimate decision-making authority and accountability [100,101]. This conceptual foundation reinforces that AI remains a tool to augment, not supplant, human expertise.

#### Mitigating Bias and Promoting Equitable Access

Despite DeepSeek’s open-weight advantage, bias and inequity remain critical challenges. Future research should conduct systematic bias audits across demographic subgroups such as sex, socioeconomic status, and ethnicity using multi-institutional and multilingual datasets [105,106]. To avoid perpetuating health care disparities, developers should expand medically validated support beyond English and Chinese to other major world languages while adapting outputs to local clinical guidelines and cultural contexts [12,107].

#### Redefining Medical Education and Workforce Development

The rapid adoption of DeepSeek demands a parallel evolution in medical curricula. Future educational interventions should cultivate “AI literacy”: the ability to critically appraise AI-generated recommendations; recognize hallucinations and bias; and integrate AI outputs with compassionate, patient-centered communication [98,108]. Institutions should develop interdisciplinary training programs that bridge clinical practice and data science to build a workforce capable of deploying, auditing, and improving medical AI systems. Finally, professional societies should establish certification and continuing education standards for AI-augmented clinical practice.

#### Unexplored Domains and Long-Term Monitoring

Most current research focuses on diagnosis, medical education, and workflow efficiency, leaving prevention and long-term care underexplored. Future investigations should prioritize disease prevention, population health management, and long-term care [103,109]. Additionally, postdeployment surveillance systems should be established to monitor real-world performance, detect emergent harms, and enable continuous model improvement, closing the loop from evidence generation to sustained safe implementation [9,90].

### Limitations of the Study

Several limitations of this study should be considered when interpreting the findings. First, the review covered literature published over a relatively short and recent timeframe. Consequently, the observed surge in publications may reflect early enthusiasm rather than sustained scientific progress. Second, although a language-agnostic search strategy was used, most included studies were published in English, with only a small number (n=13) in Chinese. This linguistic imbalance, coupled with the predominance of contributions from researchers based in China, indicates a notable geographical concentration of the available evidence. As a result, the findings may not be directly generalizable to health care systems operating within different regulatory, cultural, or infrastructural contexts. Third, the included studies exhibited substantial heterogeneity in methodologies, medical specialties, evaluation metrics, comparator models, and DeepSeek model versions—for example, R1 versus V3, which differ in parameter counts, training data, and reasoning depth. This variability precluded quantitative synthesis of outcomes and hindered direct cross-study comparisons. Although we reported version-specific findings where available, direct comparisons of performance should be interpreted with caution. Future research should adopt standardized version reporting and benchmark against fixed model checkpoints to enhance comparability and reproducibility. Finally, much of the evidence is derived from benchmarking studies, simulated cases, or retrospective analyses, with a formal quality appraisal showing that 66.6% (235/353) of included original articles were of low quality and only 6.8% (24/353) met the criteria to be considered high quality. Prospective clinical trials or RCTs assessing DeepSeek’s impact on tangible patient health outcomes in real-world clinical settings remain notably scarce. Consequently, the overall quality of the evidence base is inherently preliminary, and the reviewed corpus carries a high risk of bias. The reported strengths of DeepSeek should be interpreted with caution, as these findings predominantly derive from low-quality, controlled, nongeneralizable settings.

### Conclusion

This integrated bibliometric and scoping review synthesized the available evidence on DeepSeek’s applications in medicine. The bibliometric analysis revealed a progressive increase in publication output from January 2025 through November 2025, with China, Turkey, and the United States as the leading contributors. Keyword co-occurrence analysis formed 7 clusters; the 3 most frequent keywords were “large language model,” “artificial intelligence,” and “patient education.”

The scoping review found that DeepSeek has been evaluated across 5 primary application domains: patient education and communication, clinical decision support and treatment planning, medical education and benchmarking, clinical workflow optimization, and medical research and data analysis. In these domains, DeepSeek demonstrated variable but often competitive performance compared with proprietary models, with documented strengths in readability of patient education materials, diagnostic accuracy in select specialties, cost-efficiency, and local deployability. Nevertheless, it should be noted that most included studies were of moderate or low quality, and the evidence base is predominantly composed of benchmarking and simulation studies, with a notable scarcity of prospective clinical trials or RCTs assessing patient-relevant outcomes. Additionally, the review identified consistent limitations, including variable performance across medical specialties, model hallucinations, ethical concerns, data privacy challenges, and regulatory gaps. Future integration will require robust prospective clinical validation, expansion of multimodal capabilities, bias mitigation strategies, human-in-the-loop governance frameworks, and equitable access strategies.

## Supplementary material

10.2196/93354 Multimedia Appendix 1 Search strategy.

10.2196/93354 Multimedia Appendix 2 Quality assessment criteria for studies included in the scoping review.

10.2196/93354 Multimedia Appendix 3 The extracted data for scoping review.

10.2196/93354 Checklist 1 PRISMA-ScR (Preferred Reporting Items for Systematic Reviews and Meta-Analyses Extension for Scoping Reviews) checklist.

## Abbreviations

AI: artificial intelligence
APY: average publication year
BIBLIO: bibliometric reviews of biomedical literature
CAD-RADS: Coronary Artery Disease Reporting and Data System
GDPR: General Data Protection Regulation
GRPO: Group Relative Policy Optimization
HIPAA: Health Insurance Portability and Accountability Act
LI-RADS: Liver Imaging Reporting and Data System
LLM: large language model
MeSH: medical subject headings
PRISMA-ScR: Preferred Reporting Items for Systematic Reviews and Meta-Analyses Extension for Scoping Reviews
RCT: randomized controlled trial
WoSCC: Web of Science Core Collection
