# Supplementary material for: Applications of DeepSeek in Medicine: Bibliometric Analysis and Scoping Review
Source: J Med Internet Res. 2026 Jun 15;28:e93354. doi: 10.2196/93354 (PMC13268639; doi:10.2196/93354)
Supplement: Multimedia Appendix 1 [file jmir-v28-e93354-s001.doc]

**Applications of DeepSeek in Medicine: Bibliometric Analysis and Scoping Review**

Haoran Zhang^1,2*^, MS; Dawei Wang^3*^, PhD; Yanliang Xu^4^, BSc; Shuming Han^2^, MS; Guangxin Wang^2^, MD, PhD

*These authors contributed equally to this work

Corresponding Author: Guangxin Wang, MD, PhD; Email: y22183@email.sdfmu.edu.cn

Multimedia Appendix 2. Search strategy.

**Web of Science Core Collection (n = 286)**

| Steps | Search Terms |
| --- | --- |
| #1 | Web of Science Categories **= (**Medicine, General & Internal) OR (Oncology) OR (Neurosciences) OR (Surgery) OR (Psychiatry) OR (Clinical Neurology) OR (Cardiac & Cardiovascular Systems) OR (Radiology, Nuclear Medicine & Medical Imaging) OR (Medicine, Research & Experimental) OR (Endocrinology & Metabolism) OR (Nursing) OR (Pediatrics) OR (Genetics & Heredity) OR (Health Care Sciences & Services) OR (Psychology, Clinical) OR (Immunology) OR (Rehabilitation) OR (Dentistry, Oral Surgery & Medicine) OR (Gastroenterology & Hepatology) OR (Obstetrics & Gynecology) OR (Orthopedics) OR (Infectious Diseases) OR (Urology & Nephrology) OR (Sport Sciences) OR (Health Policy & Services) OR (Engineering, Biomedical) OR (Nutrition & Dietetics) OR (Respiratory System) OR (Toxicology) OR (Peripheral Vascular Disease) OR (Hematology) OR (Ophthalmology) OR (Dermatology) OR (Pathology) OR (Geriatrics & Gerontology) OR (Anesthesiology) OR (Otorhinolaryngology) OR (Critical Care Medicine) OR (Substance Abuse) OR (Rheumatology) OR (Behavioral Sciences) OR (Emergency Medicine) OR (Materials Science, Biomaterials) OR (Medical Informatics) OR (Integrative & Complementary Medicine) OR (Virology) OR (Reproductive Biology) OR (Allergy) OR (Audiology & Speech-Language Pathology) OR (Medical Laboratory Technology) OR (Transplantation) OR (Primary Health Care) OR (Tropical Medicine) OR (Medical Ethics) OR (Medicine, Legal) OR (Neuroimaging) OR (Andrology) OR (Public, Environmental & Occupational Health) OR (Pharmacology & Pharmacy) |
| #2 | Topic = “disease*” OR “illness*” OR “disorder*” OR “medicine*” OR “medical*” OR “hospital*” OR “clinical*” OR “health*” OR “healthcare” OR “health care” OR “treatment*” OR “Therap*” OR “medication*” OR “drug*” OR “Pharmac*” OR “Pharmacolog*” OR “Anesthesia” OR “Analgesia” OR “Nurse*” OR “Nursing*” OR “diagnos*” OR “prognos*” OR “rehabilitation*” OR “Acupuncture” OR “Biomedical Engineering” OR “Chiropractic” OR “Dentistry” OR “Dental” OR “Surger*” OR “surgical*” OR “Evidence-Based Practice*” OR “Nutrition*” OR “Optometr*” OR “Orthoptic*” OR “Podiatry” OR “Serology” OR “Toxicolog*” |
| #3 | #1 OR #2 |
| #4 | Topic = “DeepSeek” |
| #5 | #3 AND #4 AND (Publication Date: 2025/01/20 to 2025/11/30) AND (Document Types: article, review) |

**Scopus (n = 356)**

| Steps | Search Terms |
| --- | --- |
| #1 | ( SUBJAREA ( MEDI ) OR SUBJAREA ( NURS ) OR SUBJAREA ( DENT ) OR SUBJAREA ( HEAL ) ) |
| #2 | ( TITLE-ABS-KEY ( disease* ) OR TITLE-ABS-KEY ( illness* ) OR TITLE-ABS-KEY ( disorder* ) OR TITLE-ABS-KEY ( medicine* ) OR TITLE-ABS-KEY ( medical* ) OR TITLE-ABS-KEY ( hospital* ) OR TITLE-ABS-KEY ( clinical* ) OR TITLE-ABS-KEY ( health* ) OR TITLE-ABS-KEY ( healthcare ) OR TITLE-ABS-KEY ( "health care" ) OR TITLE-ABS-KEY ( treatment* ) OR TITLE-ABS-KEY ( Therap* ) OR TITLE-ABS-KEY ( drug* ) OR TITLE-ABS-KEY ( medication* ) OR TITLE-ABS-KEY ( Pharmac* ) OR TITLE-ABS-KEY ( Pharmacolog* ) OR TITLE-ABS-KEY ( Anesthesia ) OR TITLE-ABS-KEY ( Analgesia ) OR TITLE-ABS-KEY ( Nurse* ) OR TITLE-ABS-KEY ( Nursing* ) OR TITLE-ABS-KEY ( Acupuncture ) OR TITLE-ABS-KEY ( "Biomedical Engineering" ) OR TITLE-ABS-KEY ( Chiropractic ) OR TITLE-ABS-KEY ( Dentistry ) OR TITLE-ABS-KEY ( Dental ) OR TITLE-ABS-KEY ( Surger* ) OR TITLE-ABS-KEY ( surgical* ) OR TITLE-ABS-KEY ( "Evidence-Based Practice*" ) OR TITLE-ABS-KEY ( Nutrition* ) OR TITLE-ABS-KEY ( Optometr* ) OR TITLE-ABS-KEY ( Orthoptic* ) OR TITLE-ABS-KEY ( Podiatry ) OR TITLE-ABS-KEY ( Serology ) OR TITLE-ABS-KEY ( Pharmac* ) OR TITLE-ABS-KEY ( diagnos* ) OR TITLE-ABS-KEY ( prognos* ) OR TITLE-ABS-KEY ( Toxicolog* ) ) |
| #3 | #1 OR #2 |
| #4 | TITLE-ABS-KEY (“DeepSeek” ) |
| #5 | #3 AND #4 AND ( PUBYEAR = 2025 AND NOT PUBDATETXT ( December 2025 ) ) AND (Document Type: article, review) |

**PubMed (n = 370)**

| Steps | Search Terms |
| --- | --- |
| #1 | ((((((((((((((((((((((((((((((((((("Diseases Category"[MeSH Terms])) OR ("Pharmaceutical Preparations"[MeSH Terms])) OR ("Pharmacologic Actions"[MeSH Terms])) OR ("Specialty Uses of Chemicals"[MeSH Terms])) OR ("Dental Materials"[MeSH Terms])) OR (Diagnosis[MeSH Terms])) OR (Therapeutics[MeSH Terms])) OR ("Anesthesia and Analgesia"[MeSH Terms])) OR ("Surgical Procedures, Operative"[MeSH Terms])) OR (Dentistry[MeSH Terms])) OR ("Mental Disorders"[MeSH Terms])) OR ("Health Care Category"[MeSH Terms])) OR (Acupuncture[MeSH Terms])) OR ("Allied Health Occupations"[MeSH Terms])) OR ("Biomedical Engineering"[MeSH Terms])) OR (Chiropractic[MeSH Terms])) OR ("Environmental Health"[MeSH Terms])) OR ("Evidence-Based Practice"[MeSH Terms])) OR ("Health Services Administration"[MeSH Terms])) OR ("Hospital Administration"[MeSH Terms])) OR ("Medical Illustration"[MeSH Terms])) OR (Medicine[MeSH Terms])) OR (Nursing[MeSH Terms])) OR ("Nursing, Practical"[MeSH Terms])) OR ("Nutritional Sciences"[MeSH Terms])) OR (Optometry[MeSH Terms])) OR (Orthoptics[MeSH Terms])) OR ("Pharmacology"[MeSH Terms])) OR ("Pharmacy"[MeSH Terms])) OR (Podiatry[MeSH Terms])) OR ("Psychology, Medical"[MeSH Terms])) OR (Serology[MeSH Terms])) OR ("Sociology, Medical"[MeSH Terms])) OR ("Specialization"[MeSH Terms])) OR ("Toxicology"[MeSH Terms]) |
| #2 | (((((((((((((((((((((((((((((((((((((disease*[Title/Abstract])) OR (illness*[Title/Abstract])) OR (disorder*[Title/Abstract])) OR (medicine*[Title/Abstract])) OR (medical*[Title/Abstract])) OR (hospital*[Title/Abstract])) OR (clinical*[Title/Abstract])) OR (health*[Title/Abstract])) OR (healthcare[Title/Abstract])) OR (health care[Title/Abstract])) OR (treatment*[Title/Abstract])) OR (Therap*[Title/Abstract])) OR (medication*[Title/Abstract])) OR (drug*[Title/Abstract])) OR (Pharmac*[Title/Abstract])) OR (Pharmacolog*[Title/Abstract])) OR (Anesthesia[Title/Abstract])) OR (Analgesia[Title/Abstract])) OR (Nurse*[Title/Abstract])) OR (Nursing*[Title/Abstract])) OR (diagnos*[Title/Abstract])) OR (prognos*[Title/Abstract])) OR (rehabilitation*[Title/Abstract])) OR (Acupuncture[Title/Abstract])) OR (Biomedical Engineering[Title/Abstract])) OR (Chiropractic[Title/Abstract])) OR (Dentistry[Title/Abstract])) OR (Dental[Title/Abstract])) OR (Surger*[Title/Abstract])) OR (surgical*[Title/Abstract])) OR (Evidence-Based Practice*[Title/Abstract])) OR (Nutrition*[Title/Abstract])) OR (Optometr*[Title/Abstract])) OR (Orthoptic*[Title/Abstract])) OR (Podiatry[Title/Abstract])) OR (Serology[Title/Abstract])) OR (Toxicolog*[Title/Abstract]) |
| #3 | #1 OR #2 |
| #4 | "DeepSeek"[Title/Abstract] |
| #5 | #3 AND #4 AND (Publication date: from 2025/1/20 to 2025/11/30) AND (Article type: original article, review) |
