# Supplementary material for: Applications of DeepSeek in Medicine: Bibliometric Analysis and Scoping Review
Source: J Med Internet Res. 2026 Jun 15;28:e93354. doi: 10.2196/93354 (PMC13268639; doi:10.2196/93354)
Supplement: Multimedia Appendix 2 [file jmir-v28-e93354-s002.doc]

**Applications of DeepSeek in Medicine: Bibliometric Analysis and Scoping Review**

Haoran Zhang^1,2*^, MS; Dawei Wang^3*^, PhD; Yanliang Xu^4^, BSc; Shuming Han^2^, MS; Guangxin Wang^2^, MD, PhD

*These authors contributed equally to this work

Corresponding Author: Guangxin Wang, MD, PhD; Email: y22183@email.sdfmu.edu.cn

**Multimedia Appendix 3.** Quality assessment criteria for studies included in the scoping review

**High quality:** Studies with a prospective design, or studies that explicitly reported external validation using an independent dataset that was not employed for prompt engineering, fine-tuning, or parameter optimization.

**Moderate quality:**  Studies that satisfied all three of the following methodological criteria: (a) a sample size of ≥30 or justified by an a priori power calculation; (b) use of an independent test set separated from any development or calibration data; and (c) direct comparison with a clinical reference standard or human expert performance. Also included in this category are studies with a clearly described design but minor methodological limitations such as single‑center design with adequate sample size but limited control for confounding.

**Low quality:** Studies with substantial limitations, including but not limited to unvalidated benchmarking, single‑center convenience samples, technical descriptions, proof‑of‑concept studies, informal comparisons, or insufficient methodological detail to permit appraisal.
